# Natural Polyphenol-Mediated Inhibition of Ferroptosis Alleviates Oxidative Damage and Inflammation in Acute Liver Injury

**DOI:** 10.34133/bmr.0167

**Published:** 2025-03-18

**Authors:** Yangjing Su, Yunong Zeng, Minjie Zhou, Meihui Liao, Ping Qin, Rong Wu, Jiaochan Han, Xiaoqi Liang, Ze Wang, Jingjing Jiang, Zhichao Yu, Xintao Huang, Kaixin Ding, Peiheng Guo, Yi He, Ying Du, Tingting Duan, Haitao Yuan, Yuewei Ge, Ali Chen, Wei Xiao

**Affiliations:** ^1^ Center for Drug Research and Development, Guangdong Provincial Key Laboratory for Research and Evaluation of Pharmaceutical Preparations, Guangdong Pharmaceutical University, Guangzhou 510006, China.; ^2^ School of Chinese Materia Medica, Guangdong Pharmaceutical University, Guangzhou 510006, China.; ^3^ Department of Organ Transplantation, Nanfang Hospital, Southern Medical University, Guangzhou 510515, China.; ^4^ School of Traditional Chinese Medicine, Southern Medical University, Guangzhou 510515, China.; ^5^ Guangzhou Women and Children’s Medical Center, Guangzhou Medical University, Guangzhou 510623, China.; ^6^ Department of Rheumatology and Immunology, The Third Affiliated Hospital, Southern Medical University, Guangzhou 510665, China.; ^7^ Consun Pharmaceutical Group, Guangzhou 510765, China.; ^8^ Key Laboratory of Glucolipid Metabolic Disorder, Ministry of Education, Guangdong Pharmaceutical University, Guangzhou 510006, China.

## Abstract

Acetaminophen (APAP) overdose has long been recognized as the main cause of drug-induced liver injury (DILI), characterized by glutathione (GSH) depletion and reactive oxygen species (ROS) accumulation, leading to ferroptosis and inflammatory responses. There is an urgent need for liver-protective agents to combat ferroptosis, modulate oxidative stress, and ameliorate inflammation. Catechin, a well-known polyphenol compound, has been shown to have antioxidant potential. However, its protective role on APAP-induced liver injury (AILI) has not been elucidated. In this study, we evaluated the modulating effects of catechin on AILI and observed that catechin attenuated liver injury by reducing inflammation. Mechanistically, catechin alleviated hepatic oxidative stress by inhibiting ROS accumulation, malondialdehyde (MDA) production, and GSH depletion. Furthermore, catechin, as a hepatic injury reparative agent, could counteract APAP-induced hepatocyte ferroptosis by activating the xCT/GPX4 pathway, and is expected to be a novel natural inhibitor of ferroptosis. Additionally, the transcriptomic results indicated that the inhibition of *Stat1* by catechin is important for the management of AILI. Inhibition of signal transducer and activator of transcription 1 (STAT1) expression, achieved through the use of the STAT1 inhibitor fludarabine in vivo and small interfering RNA (siRNA) in vitro, was confirmed to attenuate APAP-induced ferroptosis. In conclusion, the present study identified a novel natural drug inhibitor of ferroptosis and revealed its mechanism of action to inhibit ferroptosis, regulate oxidative stress, and ameliorate inflammation in AILI. This further provides new insights into the novel natural ferroptosis inhibitors for the treatment of ROS-related inflammatory diseases.

## Introduction

Acute liver failure (ALF) is the major cause of liver-related deaths worldwide, with drug-induced liver injury (DILI), particularly from acetaminophen (APAP) overdose, being the principal trigger [1]. As a commonly used analgesic and antipyretic drug, APAP is safely administered at recommended doses [2]. However, in the event of an overdose, APAP can induce severe liver injury, even leading to ALF [3]. APAP overdose is reportedly the major cause of ALF in Western countries [4,5]. A normal dose of APAP is mainly metabolized by sulfotransferases and glucuronosyltransferases and subsequently excreted in the urine [6]. However, in increased APAP concentration, a greater portion of it is converted into the toxic intermediate N-acetyl-p-benzoquinone imine (NAPQI) by cytochrome P450 enzymes (CYPs). Excess NAPQI rapidly deletes glutathione (GSH), leading to the covalent binding of unbound NAPQI to mitochondrial proteins and the formation of APAP adducts, which induce oxidative stress and ferroptosis [7,8]. N-acetylcysteine (NAC) is the only therapeutic drug for APAP-induced liver injury (AILI); however, its clinical applications are limited due to its narrow therapeutic window and potential to trigger side effects [9]. Thus, there is an urgent need to discover new therapeutic agents and targets for alleviating AILI.

Ferroptosis is a novel iron-dependent form of programmed cell death, distinct from other cell deaths including apoptosis, pyroptosis, and necrosis [10]. It is universally known that ferroptosis is characterized by a fatal iron-dependent buildup of reactive oxygen species (ROS), which drive the peroxidation of lipids. Meanwhile, GSH is the most abundant endogenous antioxidant, neutralizing ROS and protecting cells against oxidative stress [11,12]. However, depletion of intracellular GSH level inactivates glutathione peroxidase 4 (GPX4), ultimately leading to overwhelming ferroptosis [13]. Inhibition of hepatic ferroptosis has been shown to ameliorate APAP-induced hepatotoxicity [14], suggesting that exploring new drugs that suppress APAP-induced ferroptosis could be an effective strategy for treating AILI.

Recent reports have claimed that natural products have shown promising prospects in the treatment of ferroptosis-related diseases due to their safety and efficacy features [15]. This encourages us to explore novel natural ferroptosis inhibitors from natural products for the treatment of AILI. Catechin, a polyphenol with potent antioxidant and anti-inflammatory activities, is particularly abundant in green tea and also exists in red wine, strawberries, black grapes, and apricots. [16,17]. Clinical trials have demonstrated that hypercholesterolemic patients who consistently drink catechin-rich tea obtain a range of benefits, including antioxidant, lipid-reducing, and liver-protecting effects [18]. Reportedly, catechin pretreatment restored tamoxifen-induced hepatic mitochondrial toxicity by inhibiting lipid peroxidation and protein oxidation [19]. It could also prevent liver injury by inhibiting the activation of nuclear factor κB (NF-κB) and suppressing the inflammatory factors in chronically alcohol-fed rats [20]. Despite recent studies highlighting the hepatoprotective potential of catechin, research on its protective effects against APAP-induced ferroptosis is currently limited.

Signal transducer and activator of transcription 1 (STAT1) is a transcriptional activator that plays a key role in cell signaling, with accumulating evidence suggesting its role in modulating various forms of cell death [21]. In the liver, STAT1 exhibits a multifaceted role, with recent research underscoring its importance in combating viral hepatitis, in modulating hepatic inflammation, and in the context of hepatocellular carcinoma [22]. Mechanistically, fludarabine, a STAT1 inhibitor, has been reported to up-regulate the expression of SLC7A11 and GPX4, thereby inhibiting interferon-γ-induced ferroptosis in ARPE-19 cells [23], implying that STAT1 may be a potential negative regulatory target for ferroptosis. However, it is unclear whether STAT1 mediates ferroptosis in AILI.

In this study, we focused on the protective effect of a new natural hepatic injury reparative agent, catechin, against AILI and its underlying mechanisms. Our findings revealed that (a) catechin markedly inhibited APAP-induced inflammatory responses; (b) after excluding the potential effects of catechin on APAP metabolic pathway and hepatocyte nuclear proliferation, we further found that catechin effectively restored hepatic antioxidant enzyme activity and inhibited ROS levels, thus against APAP-induced oxidative stress; (c) catechin effectively resisted APAP-induced ferroptosis by increasing the expression of GPX4 and xCT proteins and decreasing hepatocyte Fe^2+^ level; (d) catechin plays a crucial role in suppressing oxidative stress and ferroptosis by suppressing the expression of STAT1, which in turn suppresses its phosphorylation process. Based on the above findings, the present study confirmed that catechin remarkably inhibited APAP-induced inflammatory response, oxidative stress, and ferroptosis, and revealed that the core mechanism of this protective effect was the inhibition of STAT1 expression. Thus, catechin, as a novel drug candidate capable of modulating the ferroptosis pathway for the treatment of AILI, opens up new directions for therapeutic strategies for AILI.

## Materials and Methods

### Animal models

Male C57BL/6J mice, aged 6 to 8 weeks and weighing 21 to 25 g, were provided by Liaoning Changsheng Biotechnology Co. Ltd. (China). All animal procedures were approved by the local animal care and use committee of Guangdong Pharmaceutical University (no. gdpulac2023313). Mice were housed in standard laboratory conditions on a 12-h light/dark cycle. The mice were orally administered 300 mg/kg APAP (Macklin, Shanghai, China) at 8:00 PM to establish an AILI model. For catechin intervention, mice were intraperitoneally injected with catechin (100 mg/kg, Macklin, Shanghai, China) dissolved in sesame oil, followed by APAP administration. For inhibitor treatment, mice were intraperitoneally injected with ferrostatin-1 (10 mg/kg, Aladdin, Shanghai, China) or fludarabine (100 mg/kg, Shanghai yuanye Bio-Technology Co., Shanghai, China) dissolved in sesame oil. All mice were anesthetized using sodium pentobarbital, and then blood and liver were obtained.

### Primary hepatocyte isolation and culture

Mouse primary hepatocytes were isolated from mice as previously described [24]. Briefly, after perfusion with a buffer solution, the liver was digested with type IV collagenase (Worthington, NJ, USA). When the liver was completely digested, the hepatocytes were inoculated into collagen-coated dishes (Corning, NY, USA) and cultured in RPMI 1640 medium (Gibco, CA, USA) supplemented with 10% fetal bovine serum and 100 U/ml penicillin/streptomycin (Gibco, CA, USA). All cells were seeded at 1 × 10^5^ cells/cm^2^ density and cultured in an incubator maintained at 37 °C with 5% CO₂. Primary hepatocytes were pretreated with catechin (50, 100, and 150 μM) for 2 h and then co-incubated with 5 mM APAP for 24 h to investigate the protective effect of catechin on primary hepatocytes. For *Stat1* silencing, primary hepatocytes were transfected with si*Stat1* for 24 h using RNAiPro Transfection Reagent (Shenzhen Mikx Biotechnology Co. Ltd., Shenzhen, China).

### Determination of ROS and cellular labile iron

To detect hepatic ROS levels, frozen liver sections were prepared and placed on glass slides. The hepatic ROS level was assessed using dihydroethidium (DHE; Thermo Fisher Scientific, MA, USA) at 37 °C for 15 min. After incubation, the sections were gently washed 3 times with phosphate-buffered saline (PBS) for 5 min each to remove unbound DHE. To measure the cellular Fe^2+^ level, frozen mice liver sections and primary hepatocytes were incubated with the FerroOrange fluorescent probe at 37 °C for 15 min. Finally, the random fields were obtained using a Leica microscope (Leica DMi8, Wetzlar, Germany).

### Quantitative reverse transcription PCR

Total RNA extraction was performed using TRIzol reagent (Invitrogen, California, USA), followed by complementary DNA (cDNA) synthesis using a reverse transcription kit (Toyobo, Osaka, Japan). Quantitative reverse transcription polymerase chain reaction (qRT-PCR) was carried out using LightCycler 96 (Roche). The relative mRNA level was normalized with *18S* ribosomal RNA using the comparative cycle threshold cycle (CT) method. All primer sequences are listed in Table S1.

### Western blotting analysis

Liver tissues and primary hepatocytes were lysed in radioimmunoprecipitation assay (RIPA) lysis buffer (Thermo Fisher Scientific, MA, USA) containing phosphatase and protease inhibitors (Beyotime, Shanghai, China). Sodium dodecyl sulfate–polyacrylamide gel electrophoresis (SDS-PAGE) was utilized to separate the protein, which was subsequently transferred onto nitrocellulose membranes (Merck, MA, USA). Subsequently, the membranes were incubated with different primary antibodies overnight at 4 °C, followed by secondary antibodies (Proteintech, Wuhan, China) for 1 h at room temperature. The primary antibodies are listed in Table S2. Finally, the protein bands were visualized and quantified using ImageJ.

### Transcriptomic analysis

Mice were injected with 100 mg/kg catechin, followed by 300 mg/kg orally of APAP for 2 h. Subsequently, primary hepatocytes were isolated from these mice. Cellular RNA was then extracted using a TRIzol reagent. The cDNA library construction and Illumina sequencing were outsourced to Biomarker Technologies Co. Ltd. (Beijing, China). Functional annotation of differentially expressed genes (DEGs) with fold change ≥ 1.5 and *P* < 0.05 was performed to obtain the Kyoto Encyclopedia of Genes and Genomes (KEGG) pathways. For further identification of the modulated target gene, we selected the top 8,000 genes based on their median absolute deviation and processed with weighted gene coexpression network analysis (WGCNA) of RStudio software, as previously described [25]. The STRING database and Cytoscape software were used to analyze the gene network diagram.

### Quantification and statistical analysis

All data were displayed as mean ± SEM and were compared using 2-tailed unpaired Student’s *t* test or one-way analysis of variance (ANOVA) with Holm–Sidak post hoc tests. *P < 0.05, **P < 0.01, ***P < 0.001. Graphical abstracts were created with BioRender. The specific catalog numbers for the experimental reagents mentioned in the text are listed in Table S3.

## Results

### Catechin shows a remarkable protective effect in AILI

**Fig. 1.**
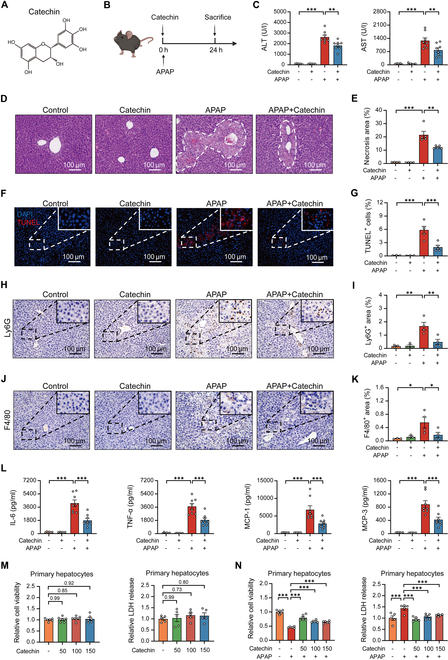
Catechin shows a remarkable protective effect in AILI. (A) Chemical structure of catechin. (B) Schematic of the experimental process. Mice were injected with catechin, followed by APAP treatment for 24 h. (C) ALT and AST serum levels (*n* = 5 to 8). (D and E) Representative H&E staining images and quantification of the necrotic areas in the liver (*n* = 4 to 5). (F and G) TUNEL staining of the liver and quantification of dead cells (*n* = 3 to 5). (H to K) Immunohistochemical staining and quantifications of Ly6G- and F4/80-positive cells in the liver (*n* = 3 to 4). (L) Quantification of cytokines and chemokines in the serum (*n* = 6 to 8). (M) CCK-8 and LDH measurements in primary hepatocytes following catechin treatment (50, 100, and 150 μM) for 24 h (*n* = 5 to 6). (N) Cell viability and LDH release in primary hepatocytes (*n* = 5 to 6). The cells were pretreated with catechin for 2 h, followed by treatment with 5 mM APAP for 24 h. Data were evaluated using one-way ANOVA with Holm–Sidak post hoc tests. *P < 0.05, **P < 0.01, and ***P < 0.001. Scale bars, 100 μm.

The AILI model was used to investigate the protective effect of catechin on APAP hepatotoxicity (Fig. 1A and B). Compared to normal mice, the ALT and AST serum levels were markedly increased in APAP-induced mice, and treatment with catechin remarkably reduced the aminotransferase levels (Fig. 1C). Histopathological examination showed that catechin administration markedly improved liver cell necrosis and hepatic inflammatory cell infiltration in APAP-treated mice (Fig. 1D to G). Catechin also decreased neutrophil and macrophage infiltrations in the liver after APAP exposure (Fig. 1H to K). In terms of inflammatory response, the mRNA levels of chemokines in APAP-treated mice were dramatically decreased after catechin administration (Fig. S1). Similarly, catechin also remarkably decreased the serum concentrations of inflammatory chemokines in APAP-treated mice (Fig. 1L). This result was further supported by the expression of MCP-1 in the liver (Fig. S2). Because primary hepatocytes are the cells of choice for determining drug toxicity in vitro [26], we established an APAP-induced primary hepatocyte injury model. We explored the protective effects of catechin against APAP-induced cytotoxicity in primary hepatocytes by assessing Cell Counting Kit-8 (CCK-8) and lactate dehydrogenase (LDH) release. Expectedly, various concentrations of catechin had no toxic effect on cell viability in primary hepatocytes and remarkably improved the relative cell viability after APAP exposure (Fig. 1M and N). In summary, these findings indicated that catechin showed its potential as a therapeutic drug for AILI by decreasing the release of inflammatory factors, reducing hepatic inflammatory cell infiltration, and protecting primary hepatocytes against APAP-induced injury.

### Catechin inhibits APAP-induced oxidative stress

**Fig. 2.**
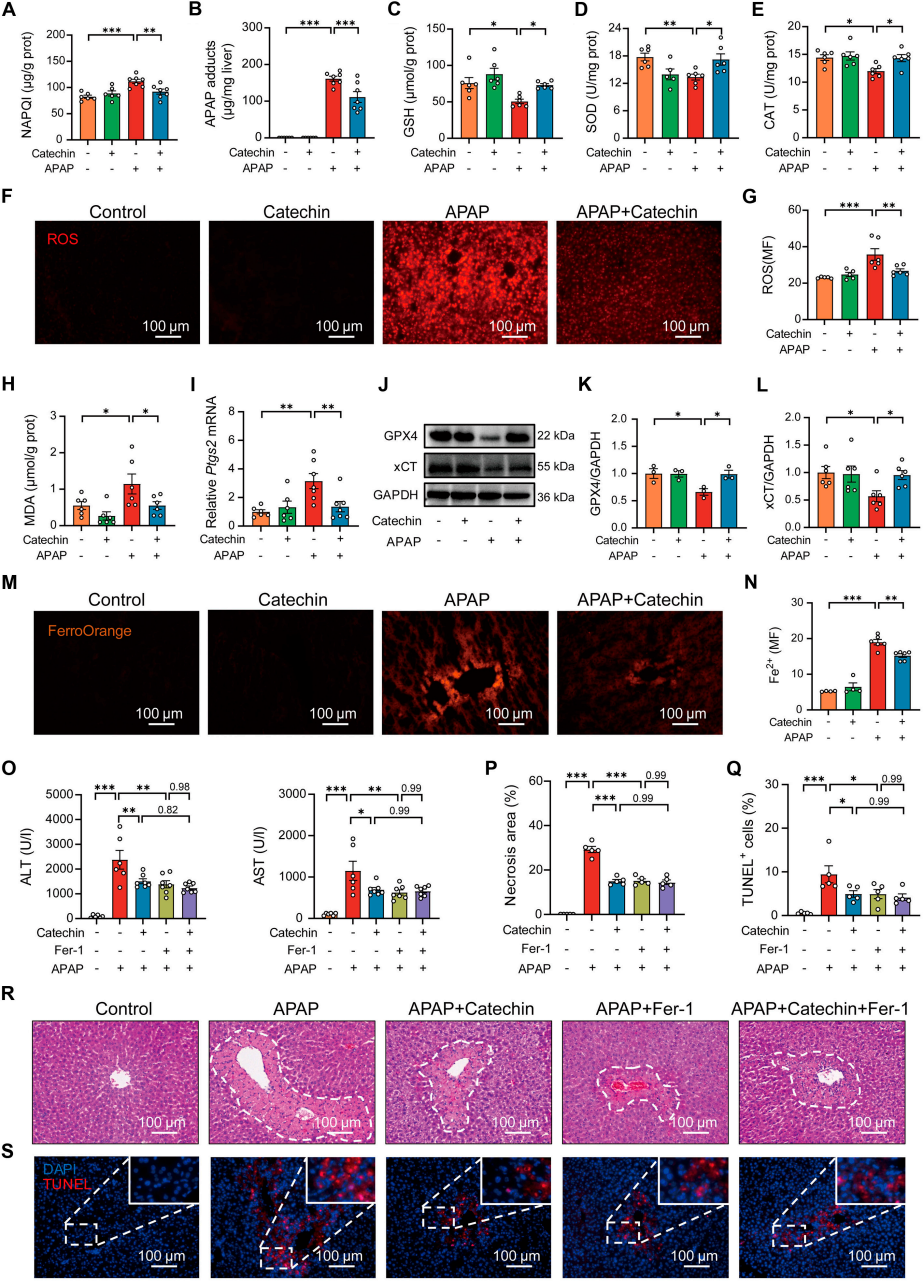
Catechin inhibits APAP-induced oxidative stress and hepatocyte ferroptosis in mice. (A) Hepatic NAPQI level in mice (*n* = 6 to 7). Mice were injected with 100 mg/kg catechin, immediately followed by APAP administration for 2 h. (B) Hepatic APAP protein adduct concentration in mice with APAP for 2 h (*n* = 6 to 7). (C to E) Hepatic GSH, SOD, and CAT levels in mice with APAP for 2 h (*n* = 5 to 6). (F and G) Intracellular ROS level in mice with APAP for 2 h (*n* = 5 to 6). (H) Hepatic MDA level in mice with APAP for 2 h (*n* = 6). (I) Relative *Ptgs2* mRNA level in the liver (*n* = 6 to 7). Mice were injected with 100 mg/kg catechin, immediately followed by APAP administration for 24 h. (J to L) Hepatic GPX4 and xCT expressions in mice with APAP for 24 h (*n* = 3 to 6). (M and N) Intracellular Fe^2+^ level in mice with APAP for 24 h (*n* = 4 to 6). (O) ALT and AST serum levels. Mice were injected with catechin or Fer-1, followed by APAP treatment (*n* = 6 to 7). (P to S) H&E- and TUNEL-stained classical plots and quantification of liver necrotic areas and cell death percentages in mice with APAP for 24 h (*n* = 5). Data were evaluated by one-way ANOVA with Holm–Sidak post hoc tests. *P < 0.05, **P < 0.01, and ***P < 0.001. Scale bars, 100 μm.

To elucidate the protective mechanism of catechin against APAP hepatotoxicity, we investigated its underlying mechanism. We first observed that the expression of hepatocyte proliferating cell nuclear antigen (PCNA) was stabilized in APAP-treated mice with or without catechin (Fig. S3). Furthermore, high-performance liquid chromatography (HPLC) analysis showed that the urine concentrations of APAP-sulf and APAP-gluc were stabilized after catechin treatment compared to the APAP group (Fig. S4). Meanwhile, catechin did not affect the expression of CYP2E1 and CYP1A2 in the liver (Fig. S5). These findings indicated that the inhibitory effect of catechin on AILI was independent of APAP metabolism and hepatocyte nuclear proliferation. Thus, we focused on the role of catechin in the regulation of APAP-induced oxidative stress. In the early stages of AILI, APAP overdose leads to a massive accumulation of NAPQI and APAP protein adducts, ultimately leading to oxidative stress [27]. We thus examined the levels of these intermediate metabolites and found that the NAPQI and APAP protein adduct levels were reduced in APAP-treated mice following treatment with catechin (Fig. 2A and B and Fig. S6). GSH, superoxide dismutase (SOD), and catalase (CAT) constitute a line of defense in the antioxidant defense system of organisms, protecting cells from oxidative damage. We then examined the levels of these antioxidant enzymes and found that catechin administration reversed the decrease in APAP-induced hepatic GSH, SOD, and CAT levels (Fig. 2C to E). We subsequently observed that the ROS accumulation was inhibited in the liver by catechin treatment after APAP exposure (Fig. 2F and G). Moreover, catechin reduced hepatic malondialdehyde (MDA) levels in APAP-treated mice (Fig. 2H), thereby inhibiting hepatic lipid peroxidation. In terms of signaling molecules, catechin treatment attenuated the enhanced phosphorylation of c-Jun N-terminal kinase (JNK) in APAP-challenged mice (Fig. S7) against hepatic mitochondrial oxidative stress. These findings indicated that the protective effect of catechin against AILI was mediated by reducing oxidative stress.

### Catechin attenuates APAP-induced hepatocyte ferroptosis

Since oxidative stress is a primary contributor to APAP-induced ferroptosis, we delved into the protective role of catechin in modulating this specific cell death pathway [28,29]. First, we detected the level of prostaglandin-endoperoxide synthase 2 (*Ptgs2*), a key marker for the onset of ferroptosis, and found that catechin could reverse the increase in *Ptgs2* level triggered by APAP (Fig. 2I). Next, we investigated the expression of GPX4 and xCT, which play pivotal roles in maintaining GSH level and preventing lipid peroxidation. Our data indicated that catechin increased the expression of GPX4 and xCT after APAP challenge (Fig. 2J to L). Furthermore, catechin treatment decreased APAP-induced hepatic Fe^2+^ accumulation (Fig. 2M and N) and similar results were observed in primary hepatocytes (Fig. S8). These findings demonstrated that catechin exerts its protective effects against APAP-induced ferroptosis by modulating key regulators of this cell death pathway, including *Ptgs2*, GPX4, and xCT, and by decreasing Fe^2+^ accumulation. To further demonstrate that catechin mediates ferroptosis in APAP-induced hepatocytes, we utilized a ferroptosis inhibitor, ferrostatin-1 (Fer-1), for validation. Catechin or Fer-1 treatment decreased the levels of alanine aminotransferase (ALT) and aspartate aminotransferase (AST) after APAP administration (Fig. 2O). Histopathological examination indicated that catechin or Fer-1 significantly reduced the areas of hepatic necrosis and the extent of cell death in mice following an APAP challenge (Fig. 2P to S). Meanwhile, serum concentrations of inflammatory chemokines were significantly reduced after catechin or Fer-1 administration (Fig. S9). In addition, catechin or Fer-1 administration effectively decreased hepatic Fe^2+^ accumulation in APAP-induced mice (Fig. S10). It is worth noting that catechin had no additional effects in Fer-1-treated mice with APAP challenge, indicating that catechin may ameliorate AILI by inhibiting ferroptosis through a similar role as Fer-1. These key findings further supported the crucial role that ferroptosis plays in the catechin treatment of APAP hepatotoxicity.

### Catechin decreases the *Stat1* gene level in APAP-treated mice

**Fig. 3.**
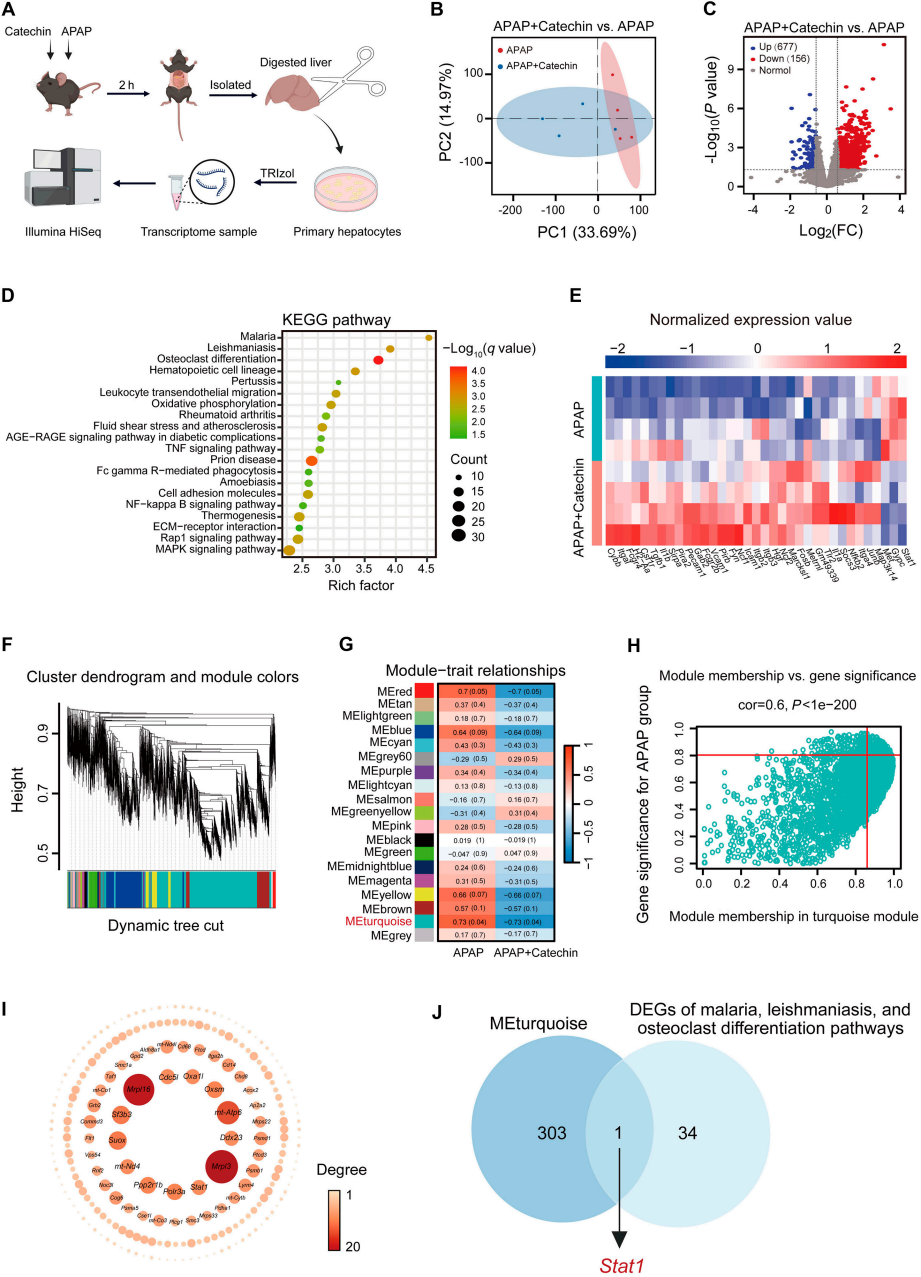
Catechin decreases *Stat1* level in APAP-treated mice. (A) Flowchart of transcriptome analysis preparation. The primary hepatocytes were isolated from APAP-treated mice with or without catechin (*n* = 4). (B and C) PCA plot and volcano plot of the transcriptomic analysis (*n* = 4). (D) KEGG pathway enrichment analysis of DEGs with fold change >1.5 (*n* = 4). (E) Heatmap of differential gene cluster analysis showing the DEGs of the top 3 pathways in the above KEGG pathway enrichment analysis (*n* = 4). (F) Hierarchical clustering dendrograms showing modules based on the coexpression topological overlap of genes (*n* = 4). (G) The relationship between module eigengenes and group traits was analyzed using Pearson’s correlation coefficient. Each cell shows its corresponding correlation and *P* value (*n* = 4). (H) Scatterplot of gene significance in the MEturquoise module (*n* = 4). (I) Network plot of the gene was obtained using the MEturquoise module definition gene significance >0.8 and module membership >0.85 as thresholds (*n* = 4). (J) Venn diagrams showing overlap of DEGs between the top 3 KEGG pathway enrichment analysis and MEturquoise module (*n* = 4).

For the in-depth exploration of specific genes mitigating APAP hepatotoxicity following catechin treatment, a transcriptome analysis was performed (Fig. 3A). Principal components analysis (PCA) plot and volcano plot showed global gene profiling between the APAP and catechin groups (Fig. 3B and C). KEGG pathway enrichment analysis was performed on DEGs with a fold change of >1.5 (Fig. 3D). DEGs from the top 3 KEGG pathways based on the enrichment factor (rich factor) were displayed in a differential gene cluster analysis heatmap, revealing that the levels of only 35 genes significantly differed between the 2 groups (Fig. 3E). For further identification of a key target that was most likely regulated by catechin, WGCNA was performed. The coexpression genes were classified into distinct modules using the dynamic tree-cut method and hierarchical agglomerative clustering (Fig. 3F and G). Since the MEturquoise module was significantly correlated between the 2 groups, we then defined the genes in this module with thresholds of gene significance >0.8 and module membership >0.85, resulting in 304 genes screened (Fig. 3H). Next, we performed a gene network analysis of the above 304 genes using Cytoscape software (Fig. 3I). Finally, the Venn diagram showed that only the *Stat1* gene overlapped with the MEturquoise module in the top 3 KEGG pathways (Fig. 3J). These findings indicated that *Stat1* is the core gene for catechin regulation of APAP hepatotoxicity.

### Catechin attenuates AILI by inhibiting STAT1 expression

**Fig. 4.**
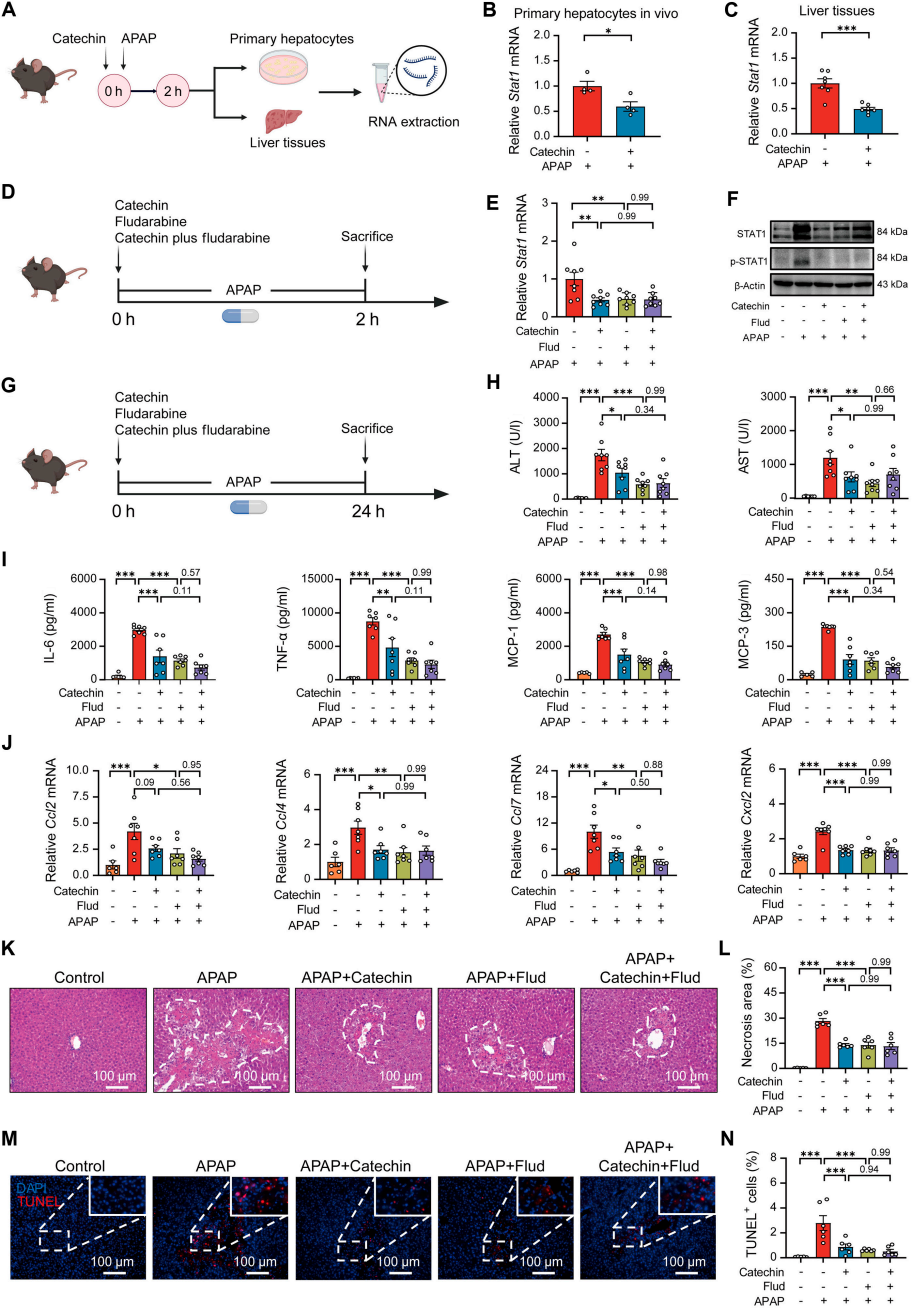
Catechin attenuates AILI by inhibiting STAT1 expression. (A to C) Schematic diagram of the experiment process and relative *Stat1* mRNA levels (*n* = 4 to 7). Mice were injected with catechin, followed by oral APAP for 2 h. The primary hepatocytes were isolated from the treated mice, and liver tissues were harvested for RNA extraction. (D) Schematic diagram of the experiment process. Mice were injected with catechin or fludarabine, immediately followed by APAP administration for 2 h. (E) Relative hepatic *Stat1* mRNA level (*n* = 8). Conditions followed the experiment process (D). (F) Hepatic STAT1 and p-STAT1 protein levels (*n* = 6). Conditions followed the experiment process (D). (G) Schematic diagram of the experiment process. Mice were injected with catechin or fludarabine, immediately followed by APAP administration for 24 h. (H) ALT and AST serum levels (*n* = 6 to 8). (I) Serum concentrations of proinflammatory cytokines (*n* = 5 to 7). (J) Relative *Ccl2*, *Ccl4*, *Ccl7*, and *Cxcl2* mRNA levels in the liver (*n* = 6 to 7). (K and L) Representative H&E staining images and quantification of liver necrotic areas (*n* = 5 to 6). (M and N) TUNEL staining of the liver and quantification of dead cells (*n* = 5 to 6). Statistical comparison was evaluated using 2-tailed unpaired Student’s *t* test (B and C) or one-way ANOVA with Holm–Sidak post hoc tests (E to N). *P < 0.05, **P < 0.01, and ***P < 0.001. Scale bars, 100 μm.

Based on transcriptomic analysis, we considered that catechin could reduce the expression of the *Stat1* gene, thereby alleviating APAP hepatotoxicity. To verify this hypothesis, we first isolated primary hepatocytes from normal mice and showed that catechin reduced *Stat1* gene expression in the cells after APAP challenge (Fig. S11). Similarly, primary hepatocytes and liver from APAP-treated mice were shown to significantly reduce *Stat1* gene expression after catechin treatment (Fig. 4A to C). Fludarabine is a nucleoside analog that causes specific depletion of STAT1 protein and mRNA but does not cause changes in other STATs [30]. Thus, we used STAT1 pharmacological inhibitor fludarabine to verify whether the function of catechin was dependent on the inhibition of STAT1. Compared to the APAP group, catechin or fludarabine significantly reduced the *Stat1* level (Fig. 4D and E). Western blot analysis further showed that catechin or fludarabine treatment inhibited STAT1 expression, as well as suppressed STAT1 phosphorylation, whereas combined treatment with catechin and fludarabine did not further reduce this expression (Fig. 4F and Fig. S12). This indicated that catechin or fludarabine showed similar effects in inhibiting the level of STAT1 in APAP-treated mice. Interestingly, catechin or fludarabine treatment decreased APAP-elevated plasma aminotransferase activities; however, catechin had no additional effects on fludarabine-treated mice with APAP challenge (Fig. 4G and H). In addition, proinflammatory factors were significantly decreased after treatment with catechin or fludarabine in APAP-treated mice (Fig. 4I and J). Furthermore, H&E (hematoxylin and eosin) and TUNEL (terminal deoxynucleotidyl transferase–mediated deoxyuridine triphosphate nick end labeling) results indicated that catechin or fludarabine could effectively reduce liver necrosis area and cell death in mice with APAP challenge (Fig. 4K to N). In conclusion, the above results indicated that catechin attenuated APAP hepatotoxicity in mice by inhibiting STAT1 expression.

### Silencing *Stat1* suppresses APAP-mediated ferroptosis in primary hepatocytes

**Fig. 5.**
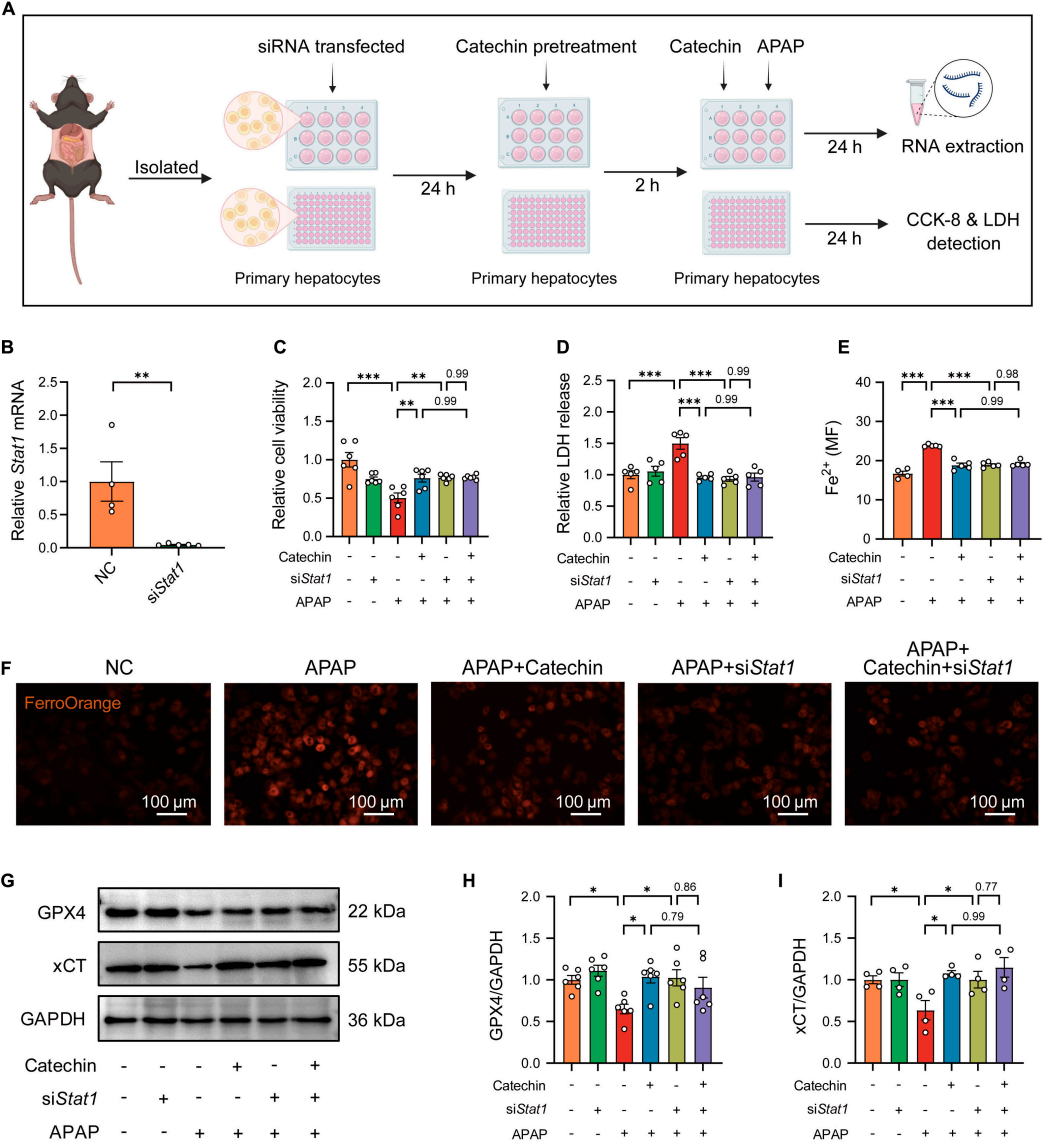
Silencing *Stat1* suppresses APAP-mediated ferroptosis in primary hepatocytes. (A) Schematic diagram of the primary hepatocytes transfected with siRNA. Primary hepatocytes were transfected with *Stat1* siRNA for 24 h and then treated with 50 μM catechin for 2 h, followed by 5 mM APAP for 24 h. (B) Relative *Stat1* mRNA in primary hepatocytes (*n* = 4 to 5). (C and D) Cell viability and LDH release in primary hepatocytes (*n* = 5 to 6). (E and F) Intracellular Fe^2+^ level was assessed in primary hepatocytes treated with APAP for 6 h (*n* = 4 to 5). (G to I) GPX4 and xCT protein levels were performed in primary hepatocytes treated with APAP for 6 h (*n* = 4 to 6). Statistical comparison was evaluated using 2-tailed unpaired Student’s *t* test (B) or one-way ANOVA with Holm–Sidak post hoc tests (C to I). *P < 0.05, **P < 0.01, and ***P < 0.001. Scale bars, 100 μm.

To explore the protective effect of inhibiting *Stat1* against APAP-induced ferroptosis, we used specific small interfering RNA (siRNA) to silence *Stat1* in APAP-treated primary hepatocytes (Fig. 5A). The qRT-PCR analysis showed that *Stat1* level was markedly reduced in primary hepatocytes after transfection with *Stat1* siRNA (Fig. 5B). As expected, catechin or *Stat1* siRNA inhibited APAP-induced cytotoxicity in primary hepatocytes, resulting in improved cell viability and reduced LDH release (Fig. 5C and D). Consistently, catechin or *Stat1* siRNA treatment reduced APAP-induced primary hepatocyte Fe^2+^ accumulation (Fig. 5E and F). In addition, catechin or *Stat1* siRNA increased the expression of GPX4 and xCT after APAP administration (Fig. 5G to I). Notably, there was no significant difference in the protective effect of catechin and si*Stat1* after APAP exposure. These in vitro results collectively support the notion that catechin ameliorated APAP-induced ferroptosis in a *Stat1*-dependent manner.

### Inhibiting STAT1 expression alleviates APAP-induced oxidative stress and hepatocyte ferroptosis

**Fig. 6.**
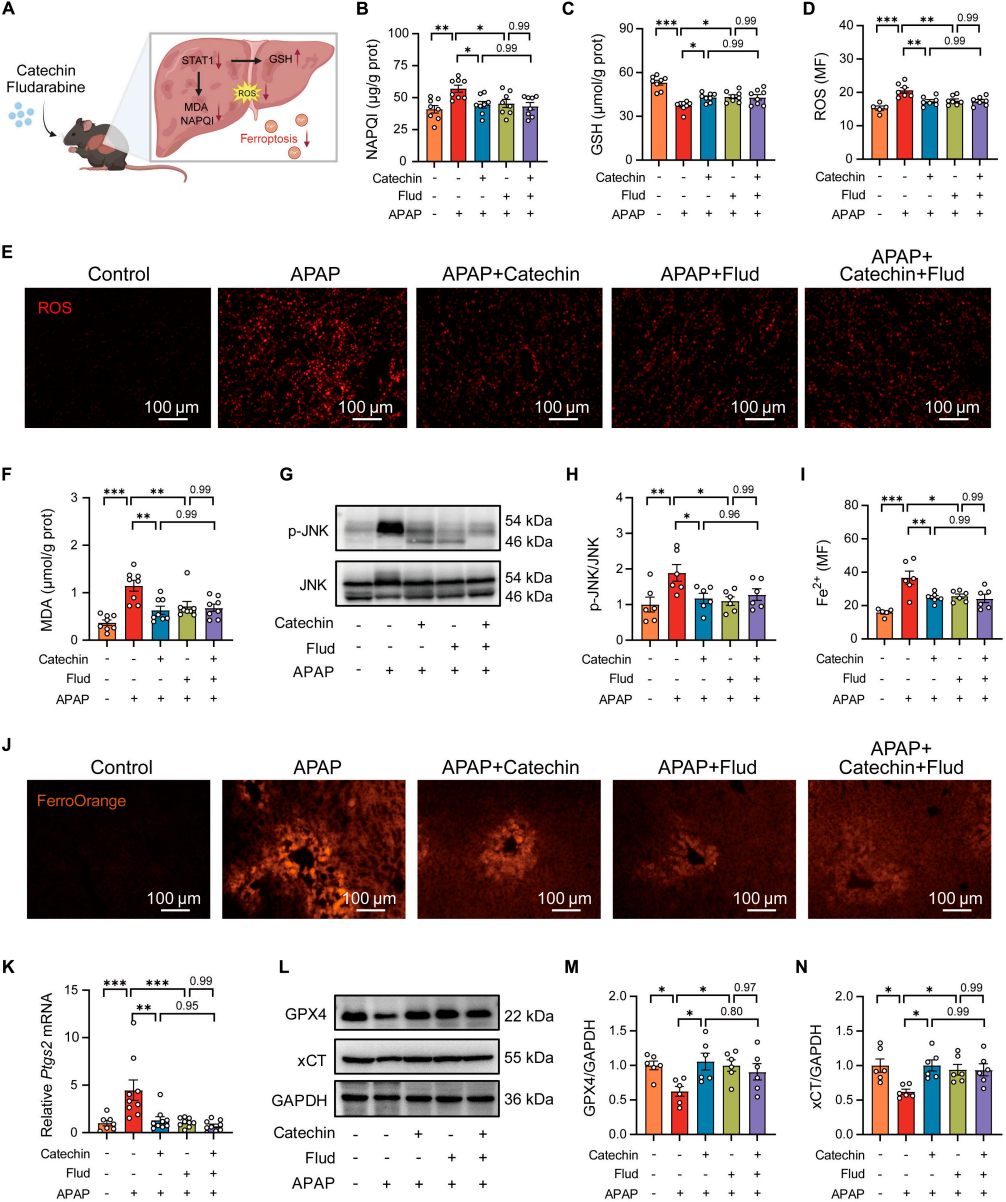
Inhibiting STAT1 expression alleviates APAP-induced hepatocyte ferroptosis in mice. (A) Inhibition of oxidative stress and ferroptosis pathways in APAP-treated mice following a reduction in hepatic STAT1 level after treatment with catechin or fludarabine. (B and C) Hepatic NAPQI and GSH levels in APAP-treated mice with catechin or fludarabine treatment for 2 h (*n* = 8). (D and E) Intracellular ROS level was assessed in APAP-treated mice with catechin or fludarabine treatment for 2 h (*n* = 5 to 7). (F) Hepatic MDA level in APAP-treated mice with catechin or fludarabine treatment for 2 h (*n* = 8). (G and H) Ratio of p-JNK/JNK in APAP-treated mice with catechin or fludarabine treatment for 2 h (*n* = 6). (I and J) Intracellular Fe^2+^ level was assessed in APAP-treated mice with catechin or fludarabine treatment for 24 h (*n* = 5 to 6). (K) Relative *Ptgs2* mRNA level in APAP-treated mice with catechin or fludarabine treatment for 24 h (*n* = 7 to 9). (L to N) GPX4 and xCT protein levels in APAP-treated mice with catechin or fludarabine treatment for 24 h (*n* = 6). Statistical comparison was evaluated using one-way ANOVA with Holm–Sidak post hoc tests. *P < 0.05, **P < 0.01, and ***P < 0.001. Scale bars, 100 μm.

After demonstrating that inhibition of *Stat1* ameliorates APAP-induced ferroptosis in primary hepatocytes, we further explored the relationship between this protective effect of APAP-induced ferroptosis in mice (Fig. 6A). First, we demonstrated that catechin or fludarabine may not influence APAP metabolism (Figs. S13 and S14). Subsequently, we observed that catechin or fludarabine was effective in reversing APAP-induced massive accumulation of NAPQI, as well as the GSH depletion (Fig. 6B and C). Catechin or fludarabine inhibited the accumulation of ROS in AILI (Fig. 6D and E). Similarly, catechin or fludarabine administration reduced the level of lipid peroxide MDA in APAP-induced mice (Fig. 6F). The augmented phosphorylation of JNK was depressed following catechin or fludarabine treatment (Fig. 6G and H). Furthermore, catechin or fludarabine administration decreased Fe^2+^ levels in APAP-induced mice (Fig. 6I and J). Moreover, we assessed the key features of ferroptosis including expression of *Ptgs2*, GPX4, and xCT. As expected, supplementation with catechin or fludarabine reversed the decrease in *Ptgs2* level and increased the expression of GPX4 and xCT to protect against AILI (Fig. 6K to N). It is worth noting that catechin failed to exhibit further protective effects cotreatment with fludarabine after APAP exposure in the above conditions. In summary, all these data highlighted the key role of STAT1 in APAP-induced ferroptosis and revealed the molecular mechanism underlying the protective role of catechin by inhibiting STAT1 expression.

## Discussion

As the main metabolic organ of the human body, the liver undertakes the metabolism of exogenous substances (including drugs and toxins) and plays an indispensable function in maintaining homeostasis [31]. However, this metabolic activity makes the liver particularly sensitive to damage from some drugs, especially APAP. In clinical practice, excessive APAP intake is a prevalent cause of DILI [32]. Notably, APAP overdose causes not only liver injury but also a specific form of cell death termed ferroptosis. Given the importance of ferroptosis in AILI, the search for drugs that can effectively inhibit the process of ferroptosis is of critical importance for the treatment of AILI.

As a treasure trove of drug discovery, natural products play an important role in preventing liver diseases [33]. Recent research has highlighted the protective effects of certain natural compounds, such as xanthohumol and kaempferol, against AILI by modulating the hepatocyte ferroptosis process [34,35]. These findings have encouraged us to discover more potent ferroptosis inhibitors from natural products. Catechin, a polyphenol with notable antioxidant properties, owes its efficacy to its polyhydroxyl structure, which neutralizes oxygen radicals and chelates metal ions [17,36]. Previous studies have demonstrated the protective effect of catechin against alcoholic liver injury and hepatic fibrosis [37,38], hinting at its therapeutic potential for liver disease management. In this study, we confirmed the protective effect of catechin against APAP-induced hepatotoxicity, which could be proved by detecting serum aminotransferase levels, evaluating hepatic inflammatory markers, and histopathological examination. However, extensive clinical trials are needed further to validate the protective effect of catechin against APAP hepatotoxicity.

In general, there is a balance between oxidative systems (including ROS, Fenton reaction, and iron ions) and antioxidant systems (including GSH, xCT, and GPX4), which antagonize each other to maintain normal physiological equilibrium within the organism and its cellular structure [39]. However, this balance is disturbed when there is excess APAP, leading to NAPQI accumulation, massive GSH depletion, and generation of large amounts of ROS, which triggers mitochondrial oxidative stress [40]. Meanwhile, JNK is activated and translocates to mitochondria during APAP hepatotoxicity, leading to mitochondrial dysfunction [41]. In this study, we found that catechin reversed APAP-induced reduction in antioxidant (GSH, SOD, and CAT) activity and accumulation of lipid peroxide (MDA). This finding suggested that catechin alleviated APAP-induced oxidative stress, which was further confirmed by the reduced accumulation of ROS and JNK phosphorylation. Given the pivotal role of oxidative stress in triggering APAP-induced ferroptosis, we hypothesized that catechin may ameliorate APAP-induced hepatic injury by modulating the ferroptosis process. Ferroptosis is a recently discovered non-apoptotic iron-dependent programmed cell death that has been regarded as a contributing factor in the development of AILI [42]. Our recent study showed that daidzein attenuated APAP-induced hepatotoxicity by modulating the *Fdps*-mediated AKT–GSK3β–Nrf2 axis to prevent ferroptosis, further underscoring the importance of inhibiting ferroptosis in the treatment of AILI [43]. GPX4 and xCT are critical regulators of anti-ferroptosis, which collectively maintain cellular antioxidant capacity [44]. Our results showed that after exposure to APAP, catechin increased the levels of GPX4 and xCT in the liver while decreasing Fe^2+^ deposition. Based on these findings, we further used Fer-1, an inhibitor of ferroptosis, for the treatment of AILI. The results showed that Fer-1 was markedly effective in ameliorating AILI, and its hepatoprotective effect was similar to catechin, which further confirmed that the liver-protecting effect of catechin was mediated by preventing ferroptosis.

Finally, an important finding of this study was that STAT1 plays a key role in treating AILI. Under normal conditions, STAT1 is localized in the cytoplasm. However, when stimulated, STAT1 undergoes phosphorylation, resulting in the formation of dimers, which are then transferred to the nucleus to function [45]. In hepatocytes, activation of STAT1 is a pro-apoptotic signal that leads to enhanced cell death and liver injury. This activation is triggered by hepatotoxic inducers, such as concanavalin A (Con A) or lipopolysaccharide (LPS) plus d-galactosamine, which promote apoptosis and inflammation, thereby aggravating liver damage [46,47]. Consistent with this finding, we observed a significant increase in STAT1 expression under the stimulation of excess APAP, and this was reversed by catechin treatment. Furthermore, inhibition of STAT1 expression by catechin or fludarabine both inhibited APAP-induced inflammation and apoptosis. On the other hand, STAT1-mediated ferroptosis has been reported in recent years in different diseases such as diabetic nephropathy, radiation-induced intestinal injury, and tumors [48,49]. Moreover, recent reports have found that down-regulation of STAT1 targets the STAT1–Nrf2 axis to inhibit crizotinib-induced hepatocellular ferroptosis, which further underscores the essential role of STAT1 as a potential therapeutic target for modulating hepatic ferroptosis [45]. In the present study, we administrated STAT1 inhibitor fludarabine as treatment in APAP-treated mice and found that fludarabine efficiently reversed APAP-induced ferroptosis. In vitro, inhibition of *Stat1* level by si*Stat1* similarly inhibited APAP-induced ferroptosis in primary hepatocytes. Based on these findings, our study illustrated that inhibition of STAT1 expression effectively suppressed APAP-induced ferroptosis, suggesting that the therapeutic effect of catechin on AILI was largely attributable to its modulation of this pathway. However, the molecular mechanism for STAT1 inhibition by catechin has not been fully elucidated, which requires more in-depth exploration in subsequent studies.

**Fig. 7.**
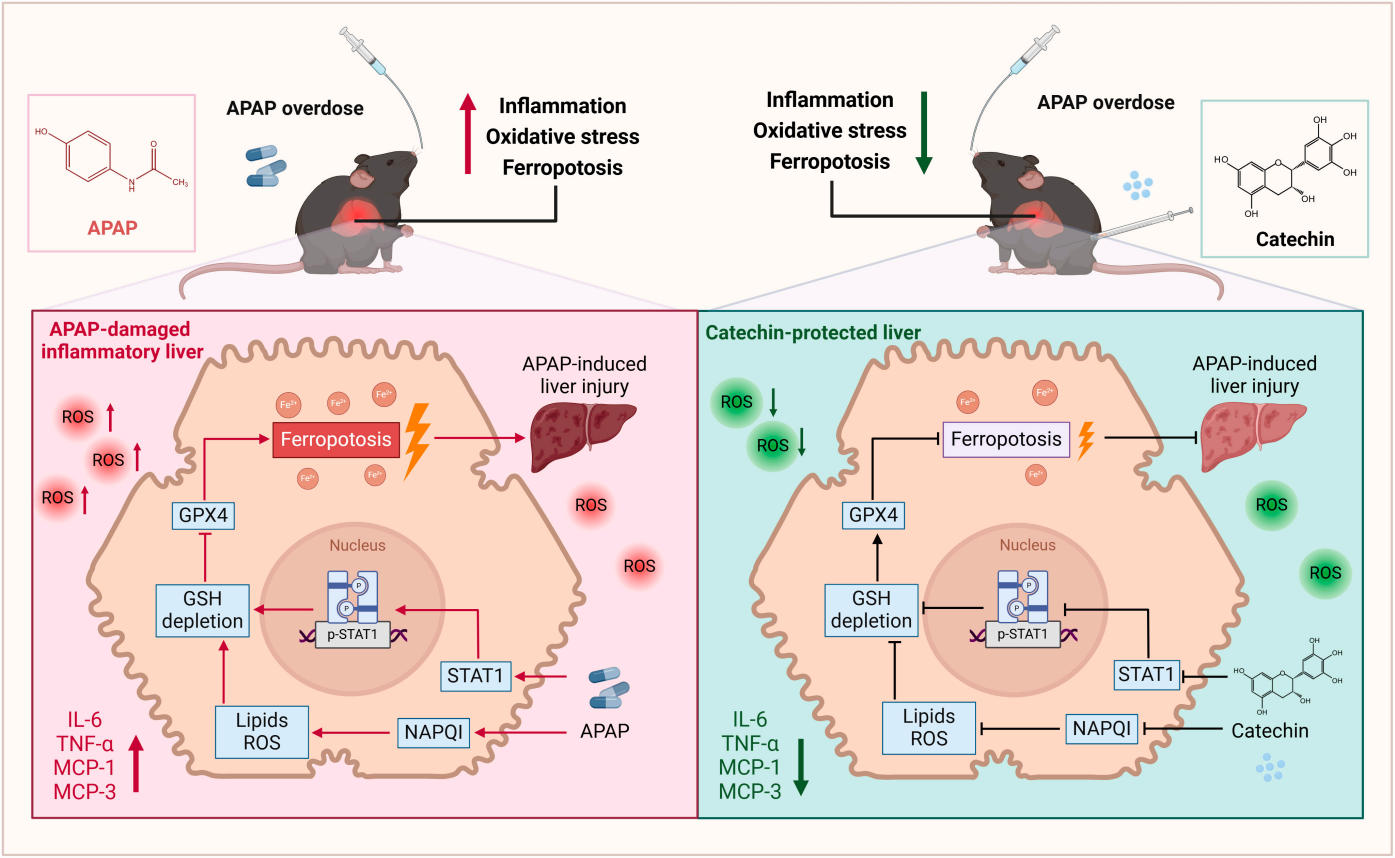
Catechin-mediated inhibition of ferroptosis alleviates oxidative damage and inflammation in APAP-induced acute liver injury.

In summary, the present study successfully revealed that catechin, as a novel natural hepatic injury reparative agent, could effectively inhibit the APAP-induced ferroptosis process, and also identified STAT1 as a novel target in this process (Fig. 7). The results of the study showed that catechin could increase hepatic antioxidant enzyme activity, protect the liver against ROS damage, and inhibit the production of APAP intermediate metabolites, thus alleviating the process of hepatic oxidative stress. Moreover, catechin activated the xCT/GPX4 pathway and inhibited the level of Fe^2+^ in the hepatocytes, further suppressing ferroptosis. In terms of molecular mechanisms, catechin ameliorated AILI by inhibiting STAT1 expression to inhibit hepatic oxidative stress, inflammation, and ferroptosis. Therefore, the present study not only developed a new natural inhibitor of ferroptosis but also provided a new drug candidate for the treatment of AILI and revealed a potential drug therapeutic target, STAT1, which opened up new avenues for the treatment of AILI.

## Data Availability

The transcriptome data were deposited in the CNGBdb: CNP0006263. The original data code is not available in this paper and can be requested from the corresponding author by email if required.
