# Supplementary material for: Natural Polyphenol-Mediated Inhibition of Ferroptosis Alleviates Oxidative Damage and Inflammation in Acute Liver Injury
Source: Biomater Res. 2025 Mar 18;29:0167. doi: 10.34133/bmr.0167 (PMC11913781; doi:10.34133/bmr.0167)
Supplement: Supplementary 1 — Materials and Methods Figs. S1 to S14 Tables S1 to S3 [file bmr.0167.f1.docx]

**Supporting information**

**Natural Polyphenol-mediated Inhibition of Ferroptosis Alleviates Oxidative Damage and Inflammation in Acute Liver Injury**

Yangjing Su^1,#^, Yunong Zeng^2,#^, Minjie Zhou^3,#^, Meihui Liao^1^, Ping Qin^4^, Rong Wu^4^, Jiaochan Han^5^, Xiaoqi Liang^4^, Ze Wang^4^, Jingjing Jiang^4^, Zhichao Yu^4^, Xintao Huang^4^, Kaixin Ding^4^, Peiheng Guo^4^, Yi He^6^, Ying Du^7^, Tingting Duan^7^, Haitao Yuan^1,^*, Yuewei Ge^2,^*, Ali Chen^1,^*, Wei Xiao^8,^*

^1^Center for Drug Research and Development, Guangdong Provincial Key Laboratory for Research and Evaluation of Pharmaceutical Preparations, Guangdong Pharmaceutical University, Guangzhou, 510006, China

^2^School of Chinese Materia Medica, Guangdong Pharmaceutical University, Guangzhou, 510006, China

^3^Department of Organ Transplantation, Nanfang Hospital, Southern Medical University, Guangzhou, 510515, China

^4^School of Traditional Chinese Medicine, Southern Medical University, Guangzhou, 510515, China

^5^Guangzhou Women and Children's Medical Center, Guangzhou Medical University, Guangzhou, 510623, China

^6^Department of Rheumatology and Immunology, The Third Affiliated Hospital, Southern Medical University, Guangzhou, 510665, China

^7^Consun Pharmaceutical Group, Guangzhou, 510765, China

^8^Key Laboratory of Glucolipid Metabolic Disorder, Ministry of Education, Guangdong Pharmaceutical University, Guangzhou, 510006, China

^#^These authors contributed equally

***Corresponding authors:** Wei Xiao (xw7688@smu.edu.cn), Ali Chen (chenali@gdpu.edu.cn), Yuewei Ge (geyuewei@gdpu.edu.cn), and Haitao Yuan (yht193525@163.com).

**Materials and methods**

**Biochemical and ELISA assays**

The plasma levels of alanine aminotransferase (ALT) and aspartate aminotransferase (AST) were determined using a commercial kit (Nanjing Jiancheng Bioengineering Institute, Nanjing, China). The hepatic levels of superoxide dismutase (SOD), catalase (CAT), and GSH were measured using reagent kits (Nanjing Jiancheng Bioengineering Institute, Nanjing, China). Malondialdehyde (MDA; Beyotime, Shanghai, China) and NAPQI (Boshen, Nanjing, China) were measured in liver using commercial assay kits. Cell viability was determined using the Cell Counting Kit-8 assay (CCK-8; Meilunbio, Dalian, China). Lactase dehydrogenase (LDH) was measured using the CytoTox 96® Non-Radioactive Cytotoxicity Assay kit (Promega, WI, USA). For measurement of serum cytokines, the plasma levels of tumor necrosis factor α (TNF-α), interleukin 6 (IL-6), and monocyte chemoattractant protein 1 (MCP-1) were determined using the corresponding commercial ELISA kits (Neobioscience, Shenzhen, China), and MCP-3 was determined using a commercial ELISA kit (Cusabio, Wuhan, China).

**Histopathological analysis and immunohistochemistry**

Livers were freshly collected and fixed in 4% paraformaldehyde for 48 h and were then embedded, sliced, and stained with hematoxylin and eosin (H&E). To assess cell death, a commercial kit (KeyGEN, Nanjing, China) was used for terminal deoxynucleotidyl transferase dUTP nick end labeling (TUNEL) staining. For immunohistochemistry (IHC) staining of Ly6G and F4/80, antigen retrieval was performed using a sodium citrate buffer. The slides were incubated in 3% H_2_O_2_ for 15 min and then with 5% BSA (Sigma-Aldrich, MO, USA) for 15 min at 37℃. Subsequently, the slides were incubated with anti-rabbit F4/80 (Abcam, MA, USA) or anti-rabbit Ly6G (Abcam, MA, USA) antibody at 4℃ overnight. Afterward, the slides were incubated with a secondary antibody (Servicebio, Wuhan, China) at 37℃ for 20 min, followed by visualization with 3,3’-diaminobenzidine and counterstaining with hematoxylin. Finally, at least 5–8 fields in each slide were randomly selected and observed under a microscope (Leica DMi8, Wetzlar, Germany).

**High-performance liquid chromatography (HPLC) analysis**

To prepare liquid phase samples, 1 ml of methanol was added for each 100 mg of liver tissue for homogenization, and 400 µl of methanol was added for each 1 µl of urine and mixed. Subsequently, the samples were centrifuged at 15,000 g for 15 min and the resulting supernatant was vacuum dried for 6 h and resuspended in methanol. For chromatographic analysis, a 10 µL sample was injected into a Cosmosil-C18 column (250 mm × 4.6 mm, 5 µm, Nacalai Tesque, Kyoto, Japan). For quantifying APAP-protein adducts, the mobile phase consisted of methanol and 0.1% (v/v) formic acid water at a volume ratio of 20:80 for 10 min. For detecting APAP sulfate (APAP-sulf, Sigma, USA) and APAP glucuronide (APAP-gluc, Sigma, USA), the mobile phase was a mixture of acetonitrile and 0.1% (v/v) formic acid water at a volume of 10:90 for 15 min. The system operated at a flow rate of 1 mL/min and a detection wavelength of 254 nm. Data acquisition and analysis were facilitated using Agilent LC1260 software.

**Quantification and statistical analysis**

All data were displayed as mean ± standard error of the mean (SEM) and were compared using two-tailed unpaired Student’s t-test or one-way ANOVA with Holm–Sidak post hoc tests. **p*<0.05, ***p*<0.01, ****p*<0.001. Create graphical abstracts with BioRender.


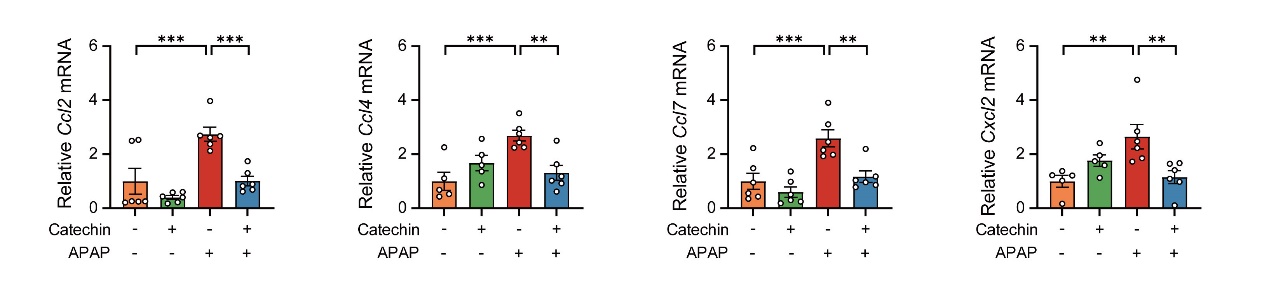


**Figure S1** Relative *Ccl2*, *Ccl4*, *Ccl7*, and *Cxcl2* mRNA levels were determined in mice with APAP for 24h (n = 5–6). Data were represented as mean ± SEM and were evaluated using one-way ANOVA with Holm–Sidak post hoc tests. ***p* < 0.01 and ****p* < 0.001.


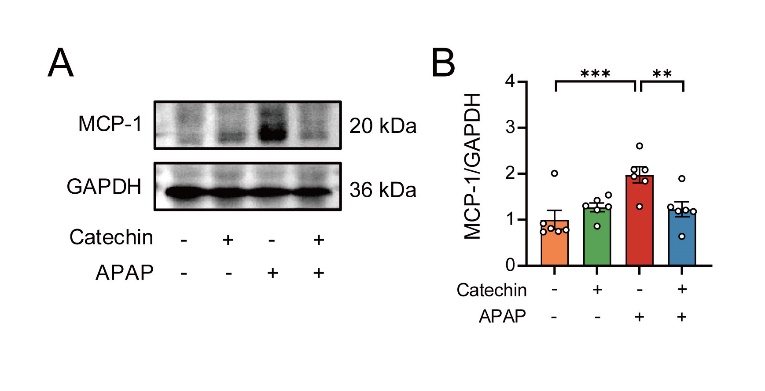


**Figure S2** Hepatic MCP-1 expression in mice with APAP for 24h (n = 6). Data were represented as mean ± SEM and were evaluated using one-way ANOVA with Holm–Sidak post hoc tests. ***p* < 0.01 and ****p* < 0.001.


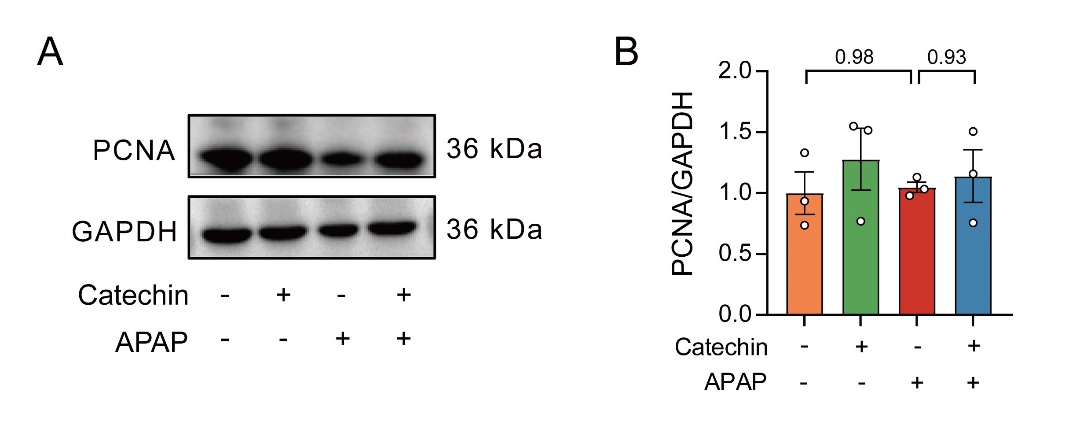


**Figure S3** Hepatic PCNA expression in mice with APAP for 24h (n = 3). Data were represented as mean ± SEM and were evaluated using one-way ANOVA with Holm–Sidak post hoc tests.


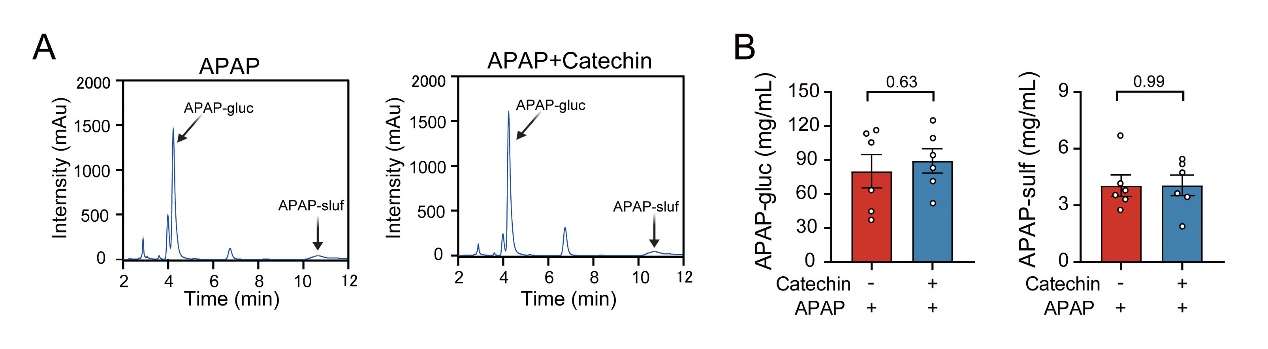


**Figure S4** Urine levels of APAP-gluc and APAP-sulf metabolite in mice with APAP for 2h (n = 6). Data were represented as mean ± SEM and were evaluated using two-tailed unpaired Student’s t-test.


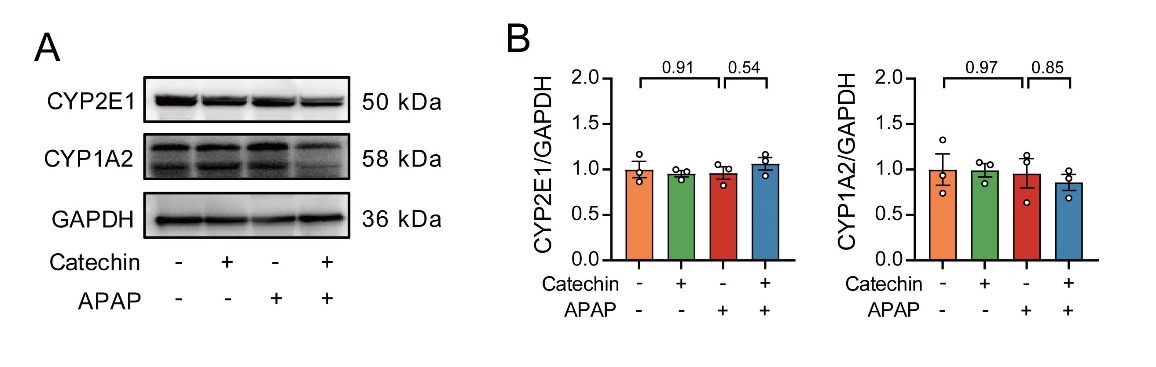


**Figure S5** Hepatic CYP2E1 and CYP1A2 expression in mice with APAP for 2h (n = 3). Data were represented as mean ± SEM and were evaluated using one-way ANOVA with Holm–Sidak post hoc tests.


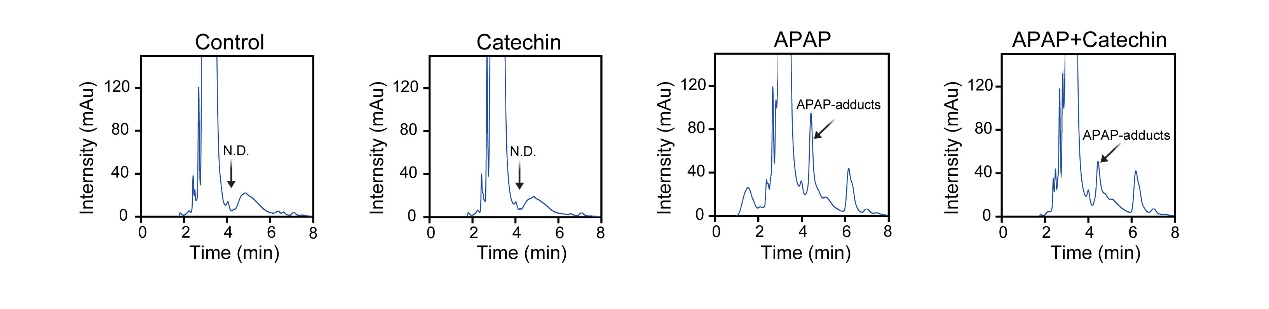


**Figure S6** Hepatic APAP-protein adduct concentration in mice with APAP for 2h.


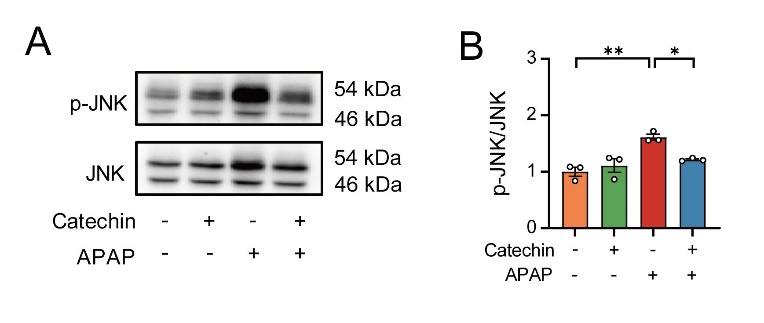


**Figure S7** Hepatic p-JNK expression in mice with APAP for 2h (n = 3). Data were represented as mean ± SEM and were evaluated using one-way ANOVA with Holm–Sidak post hoc tests. **p* < 0.05 and ***p* < 0.01.


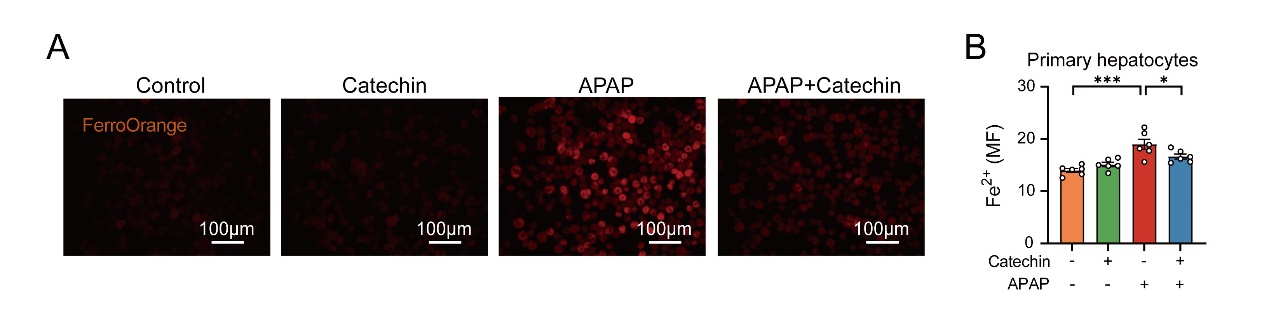


**Figure S8** Intracellular Fe^2+^ level was assessed in primary hepatocytes pretreated with 50 μM catechin for 2 h, followed by treatment with 5 mM APAP for 6 h (n = 6). Data were represented as mean ± SEM and were evaluated using one-way ANOVA with Holm–Sidak post hoc tests. **p* < 0.05 and ****p* < 0.001. Scale bars, 100 μm.


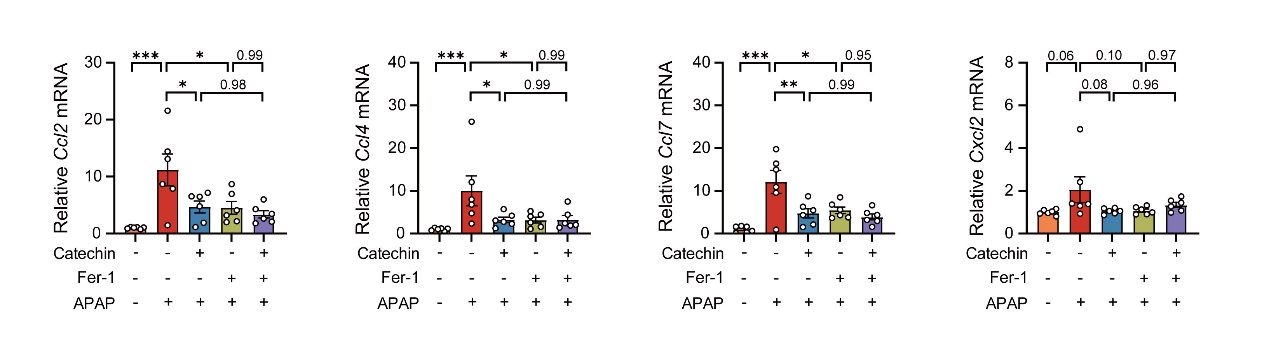


**Figure S9** Relative *Ccl2*, *Ccl4*, *Ccl7*, and *Cxcl2* mRNA levels were determined in mice with APAP for 24h (n = 6). Data were represented as mean ± SEM and were evaluated using one-way ANOVA with Holm–Sidak post hoc tests. **p* < 0.05, ***p* < 0.01, and ****p* < 0.001.


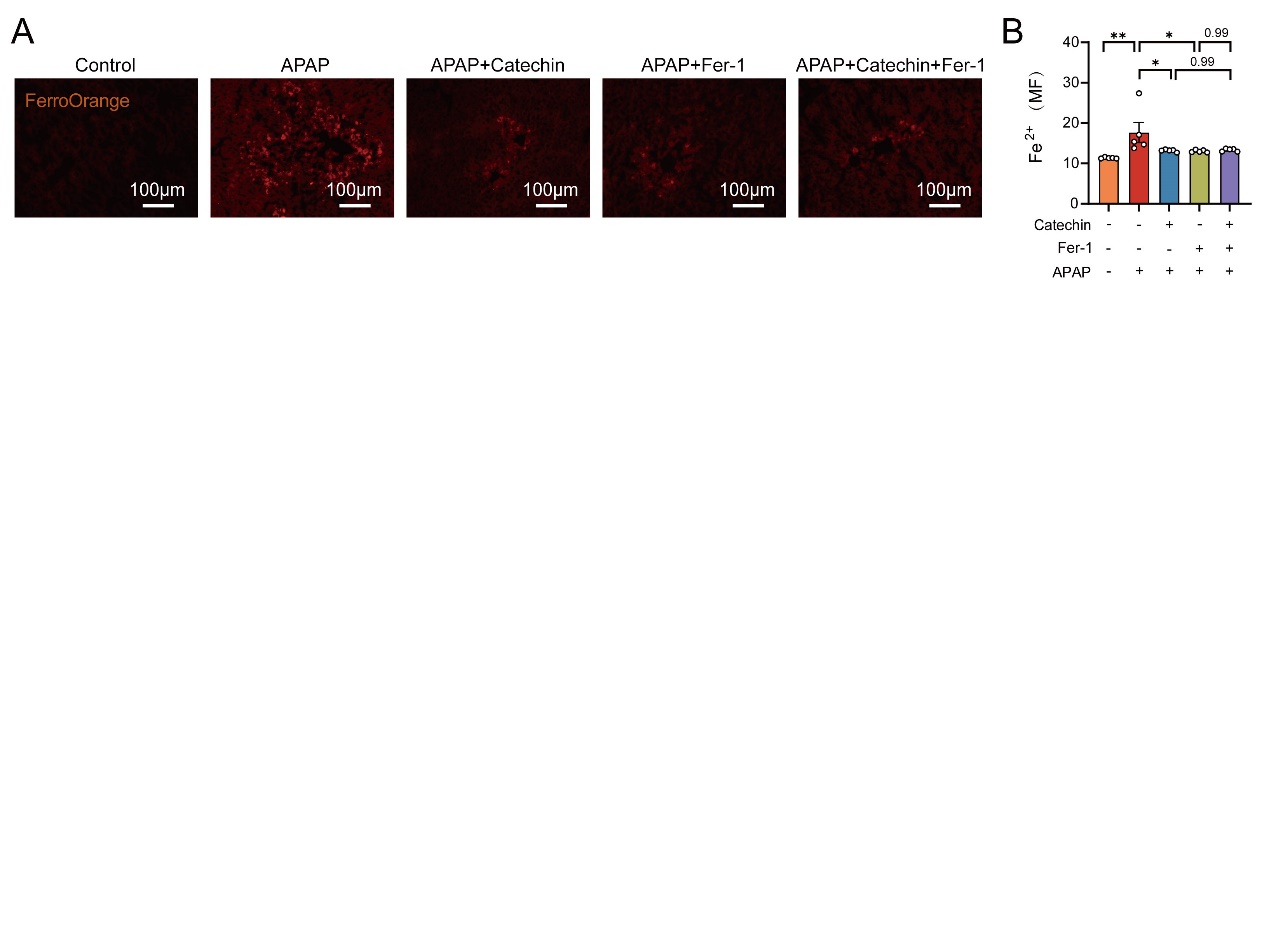


**Figure S10** Intracellular Fe^2+^ level was assessed using a FerroOrange fluorescent probe in APAP-treated mice with catechin or Fer-1 treatment for 24 h (n = 5). Data were represented as mean ± SEM and were evaluated using one-way ANOVA with Holm–Sidak post hoc tests. **p* < 0.05 and ***p* < 0.01. Scale bars, 100 μm.


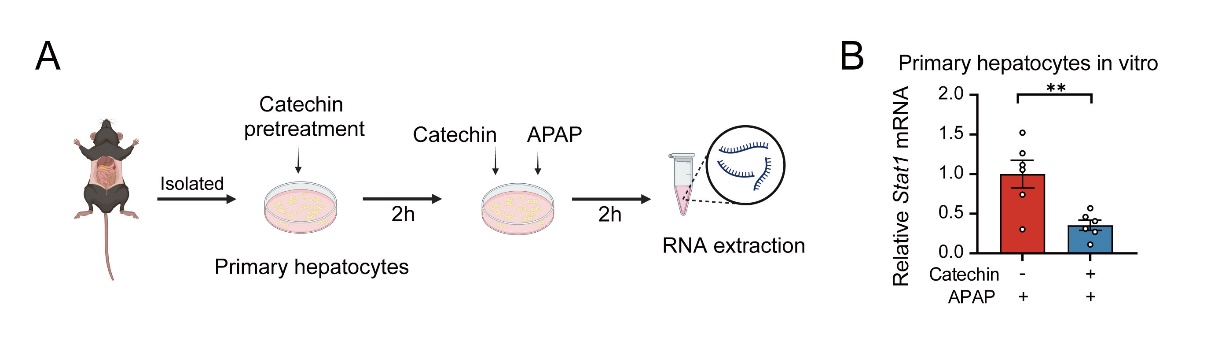


**Figure S11** (A) Schematic diagram of the in vitro experiment. Primary hepatocytes were isolated from normal mice and then pretreated with or without 50 µM catechin for 2 h, followed by treatment with 5 mM APAP for 2 h. (B) Relative *Stat1* mRNA level was determined from primary hepatocytes in vitro experiment (n = 6). Data were represented as mean ± SEM and were evaluated using two-tailed unpaired Student’s t-test. ***p* < 0.01.


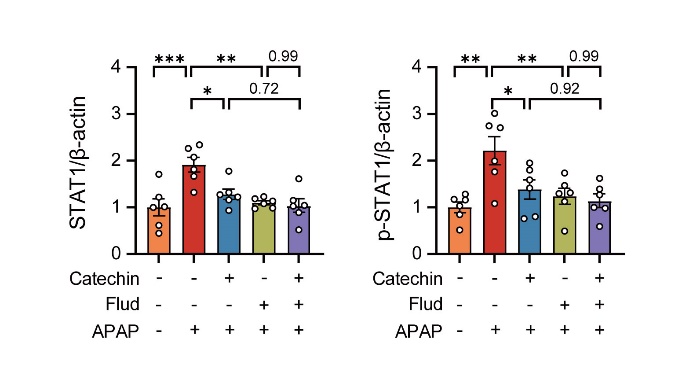


**Figure S12** Hepatic STAT1 and p-STAT1 protein levels in APAP-treated mice with catechin or fludarabine treatment for 2 h (n = 6). Data were represented as mean ± SEM and were evaluated using one-way ANOVA with Holm–Sidak post hoc tests. **p* < 0.05, ***p* < 0.01, and ****p* < 0.001.


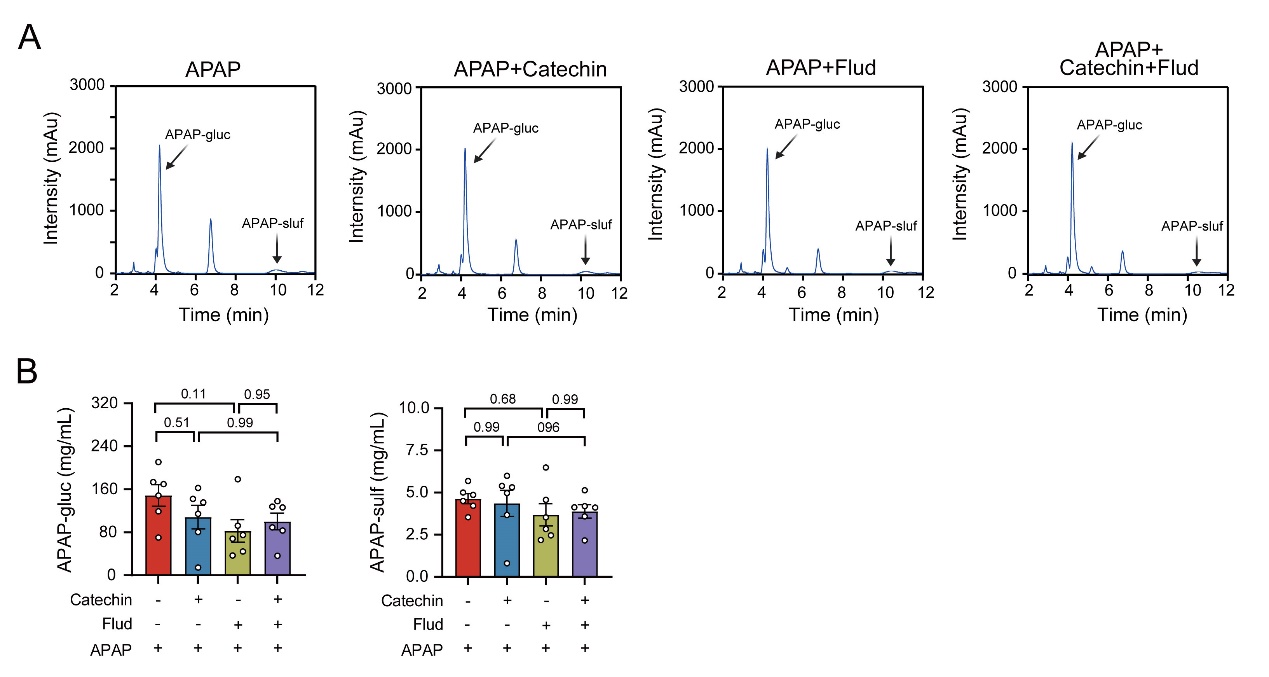


**Figure S13** APAP-gluc and APAP-sulf metabolite levels. Mice were injected with 100 mg/kg catechin or 100 mg/kg fludarabine, immediately followed by APAP for 2 h (n = 6). Data were represented as mean ± SEM and were evaluated using one-way ANOVA with Holm–Sidak post hoc tests.


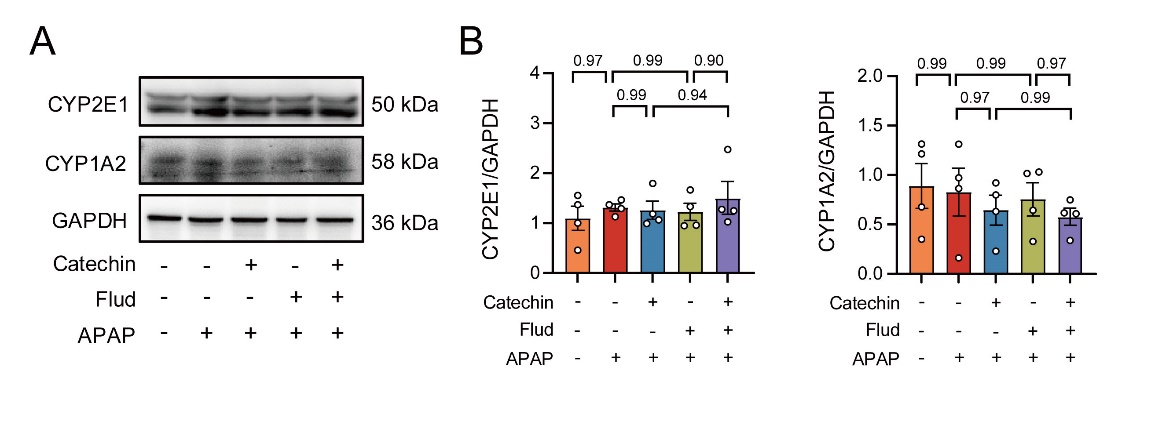


**Figure S14** Hepatic CYP2E1 and CYP1A2 expressions (n = 4). Data were represented as mean ± SEM and were evaluated using one-way ANOVA with Holm–Sidak post hoc tests.

**Table S1**

**Primers for qRT‑PCR**

| **Name** | **Forward primer (5’-3’)** | **Reverse primer (5’-3’)** |
| --- | --- | --- |
| ***18S*** | CGATCCGAGGGCCTCACTA | AGTCCCTGCCCTTTGTACACA |
| ***Stat1*** | GCTGCCTATGATGTCTCGTTT | TGCTTTTCCGTATGTTGTGCT |
| ***Ccl2*** | TAAAAACCTGGATCGGAACCAAA | GCATTAGCTTCAGATTTACGGGT |
| ***Ccl4*** | TTCCTGCTGTTTCTCTTACACCT | CTGTCTGCCTCTTTTGGTCAG |
| ***Ccl7*** | CCACATGCTGCTATGTCAAGA | ACACCGACTACTGGTGATCCT |
| ***Cxcl2*** | CCAACCACCAGGCTACAGG | GCGTCACACTCAAGCTCTG |
| ***Ptgs2*** | TTCCAATCCATGTCAAAACCGT | AGTCCGGGTACAGTCACACTT |

**Table S2**

**Antibody information**

| **Antibodies** | **Sources** | **Identifier** | **Dilution ratios** |
| --- | --- | --- | --- |
| Anti-rabbit F4/80 | Abcam | Cat# ab111101 | 1:1000 |
| Anti-rabbit Ly6G | Abcam | Cat# ab238132 | 1:2000 |
| MCP-1 polyclonal antibody | Proteintech | Cat# 26161-1-AP | 1:1000 |
| PCNA antibody | Abmart | Cat# P60051 | 1:1000 |
| Cytochrome P450 1A1/2 antibody | Affinity | Cat# AF5312 | 1:2000 |
| CYP2E1-specific polyclonal antibody | Proteintech | Cat# 19937-1-AP | 1:2000 |
| SAPK/JNK antibody | Cell Signaling Technology | Cat# 9252 | 1:1500 |
| Phospho-SAPK/JNK (Thr183/Tyr185) antibody | Cell Signaling Technology | Cat# 9255 | 1:3000 |
| GPX4 antibody | Affinity | Cat# DF6701 | 1:1000 |
| xCT antibody | Abmart | Cat# T57046 | 1:1000 |
| STAT1 antibody | Abmart | Cat# T55227 | 1:1000 |
| Phospho-STAT1(Tyr701) antibody | Abmart | Cat# TP56498 | 1:1000 |
| Beta actin monoclonal antibody | Affinity | Cat# 66009-1-Ig | 1:5000 |
| GAPDH polyclonal antibody | Affinity | Cat# 10494-1-AP | 1:5000 |
| Anti-rabbit IgG, HRP-linked antibody | Cell Signaling Technology | Cat# 7074 | 1:10000 |
| HRP-conjugated Affinipure Goat Anti-Mouse IgG(H+L) | Proteintech | Cat# SA00001-1 | 1:10000 |

**Table S3**

**Key resources**

| **Chemicals** | **Source** | **Identifer** |
| --- | --- | --- |
| Acetaminophen | Macklin | Cat# A800441 |
| Catechin | Macklin | Cat# C823329 |
| Fludarabine | Shanghai yuanye Bio-Technology Co. | Cat# S44591 |
| Ferrostatin-1 | Aladdin | Cat# F129882 |
| Acetaminophen sulfate potassium salt | Sigma | Cat# UC448 |
| Acetaminophen glucuronide | Sigma | Cat# 43073 |
| Sesame oil | Shanghai yuanye Bio-Technology Co. | Cat# S27343 |
| Collagenase types IV | Worthington | Cat# LS004188 |
| PBS | Gibco | Cat# C10010500BT |
| Collagen I | BD PharMingen | Cat# 354236 |
| RPMI1640 medium | Gibco | Cat# C11875 |
| Fetal bovine serum | Gibco | Cat# 10270-106 |
| Penicillin/streptomycin | Gibco | Cat# 15140122 |
| Bovine serum albumin | Sigma | Cat# V900933 |
| Non-Fat powdered milk | Solarbio | Cat# D8340 |
| TRIzol reagent | Invitrogen | Cat# 15596018 |
| RIPA lysis buffer | Beyotime | Cat# P0013B |
| Primary antibody dilution buffer | Beyotime | Cat# P0023A |
| Nitrocellulose membranes | Merck | Cat# HATF00010 |
| Dihydroethidium | Thermo Scientific | Cat# D23107 |
| FerroOrange fluorescent probe | Dojindo | Cat# F374 |

| **Commercial Assays** | **Source** | **Identifer** |
| --- | --- | --- |
| Hoechst 33258 | Beyotime | Cat# C1011 |
| ECL Western Blotting Substrate | Biosharp | Cat# BL520B |
| Multicolor Prestained Protein Ladder | Epizyme Biomedical Technology | Cat# WJ102 |
| ALT assay kit | Nanjing Jiancheng Bioengineering Institute | Cat# C009-3-1 |
| AST assay kit | Nanjing Jiancheng Bioengineering Institute | Cat# C010-2-1 |
| SOD assay kit | Nanjing Jiancheng Bioengineering Institute | Cat# A001-3-2 |
| CAT assay kit | Nanjing Jiancheng Bioengineering Institute | Cat# A007-1-1 |
| GSH assay kit | Nanjing Jiancheng Bioengineering Institute | Cat# A006-2-1 |
| MDA assay kit | Beyotime | Cat# S0131S |
| NAPQI assay kit | Boshen | Cat# BS-E10517M1 |
| CytoTox 96® Non-Radioactive Cytotoxicity Assay kit | Promega | Cat# G1780 |
| Cell Counting kit-8 | Meilunbio | Cat# MA0218 |
| RNAiPro Transfection Reagent | Shenzhen Mikx Biotechnology Co., Ltd. | Cat# MK4018 |
| Mouse IL-6 ELISA kit | Neobioscience | Cat# EMC004 |
| Mouse TNF-α ELISA kit | Neobioscience | Cat# EMC102a |
| Mouse MCP-1 ELISA kit | Neobioscience | Cat# EMC113 |
| Mouse MCP-3 ELISA kit | Cusabio | Cat# CSB-E07426m |
| TUNEL Assay kit | KeyGEN | Cat# KGA7063 |
| Reverse transcription reagent kit | Toyobo | Cat# FSQ-101 |
| SYBR Green Master Mix | Toyobo | Cat# QPK-201C |
| BCA Protein Assay Kit | GLPBIO | Cat# GK10009 |

| **Oligonucleotides** | | **Sense strand** | **Antisense strand** |  |
| --- | --- | --- | --- | --- |
| si-*Stat1* | GCUGUUACUUUCCCAGAUAUU | | AAUAUCUGGGAAAGUAACAGC | |
| si-negative control | UUCUCCGAACGUGUCACGUTT | | ACGUGACACGUUCGGAGAATT | |

| **Software and algorithms** | | |
| --- | --- | --- |
| ImageJ software | NIH | https://imagej.nih.gov/ij/ |
| R studio software | RStudio | https://www.rstudio.com/ |
| GraphPad Prism 8 software | GraphPad | https://www.graphpad.com/scientific-software/prism/ |
| Adobe Illustrator 2021 | Adobe | https://www.adobe.com/ |
| Agilent LC1260 software | Agilent Technologies | https://www.agilent.com/ |
| BioRender | BioRender | https://biorender.com/ |
| STRING | STRING | https://string-db.org/ |
| Cytoscape software | Cytoscape | https://cytoscape.org/ |
